# Avidity Studies in *Anisakis simplex*-Associated Allergic Diseases

**DOI:** 10.1155/2013/106781

**Published:** 2013-05-23

**Authors:** Carmen Cuéllar, Ana Valls, Consolación de Frutos, Marta Rodero, Alvaro Daschner

**Affiliations:** ^1^Departamento de Parasitología, Facultad de Farmacia, Universidad Complutense, 28040 Madrid, Spain; ^2^Servicio de Alergia, Instituto de Investigación Sanitaria, Hospital Universitario de la Princesa, C/Diego de León 62, 28006 Madrid, Spain

## Abstract

Gastroallergic anisakiasis (GAA) and *Anisakis*-sensitization-associated chronic urticaria (CU+) differ with respect to specific IgE levels. We hypothesised different immunoglobulin avidities in both entities as well as their dependence on TI and fish consumption. 16 patients with GAA and 17 patients with CU+ were included, and immunoglobulin levels were analysed by CAP (Phadia). IgE and IgG avidity indexes (AvIgE and AvIgG, resp.) were also determined. IgG avidity was higher in GAA than in CU+ (*P* = 0.035), whereas there was a tendency to lower IgE avidity in GAA (*P* = 0.095). When analysing all patients, AvIgG was positively correlated with specific IgE, IgG, and IgG_4_ as well as total IgE (Rho between 0.66 and 0.71; *P* < 0.002), but AvIgE was negatively correlated with specific IgE (Rho −0.57; *P* < 0.001), specific IgG_4_ (Rho −0.38; *P* < 0.05), and total IgE (Rho 0.66; *P* < 0.001). In GAA, weekly fish consumption was positively associated with AvIgE (Rho 0.51; *P* = 0.05). A multivariate regression showed that time interval was the main explaining factor for AvIgE in GAA. We could show a differential behaviour of immunoglobulin isotype avidities in both entities and their dependence on fish-eating habits as well as on the time elapsed to the last parasitic episode.

## 1. Introduction

*Anisakis simplex* is a fish parasite of worldwide distribution, whose third stage larva can be present in a huge number of marine teleost fishes. Humans can therefore be accidental hosts by consuming crude or undercooked fish [1]. Whereas IgE production is an evolutionary-maintained immunologic feature in mammals and thus can also be verified in all cases of human parasitic forms by this nematode, the allergenic potential has been an emerging health concern in the last years, giving rise to a large number of studies searching for proteins able to stimulate a specific IgE response [2, 3]. Gastroallergic anisakiasis has been described as a differential entity, where an acute parasitism by *A. simplex* produces also a clinically relevant IgE-mediated hypersensitivity reaction with appearance of acute urticaria, angioedema, or anaphylaxis [4].

It is well known that, in GAA, specific as well as total IgE and other specific immunoglobulin isotype levels depend upon the time interval elapsed (TI) between the acute parasitic episode and the obtaining of the serum sample [5]. The parasite is not adapted to the human environment and neither survives nor moults to its final stage. Even if the parasitic episode is always short-lived for mainly some hours, but maximally for a few days, as has been documented by gastroscopic findings in GAA, the immune stimulation, as witnessed by its humoral response, reaches its peak after four to six weeks and IgE is detectable in human sera or can be shown by skin prick tests (SPT) for more than ten years [5].

Another clinical entity, where a previous acute parasitic episode is suspected to play at least a necessary factor in a disease of multifactorial genesis, has been proposed to be an *Anisakis* sensitization-associated chronic urticaria (CU+) and has been described as a different phenotype of urticaria with distinct clinical as well as immunologic features with respect to other chronic urticaria forms, but also compared to GAA [6–9]. Furthermore, association of chronic urticaria with *A. simplex* hypersensitivity is now reported from different geographic regions [10]. Therefore, even if current bibliography shows chronic urticaria to have a frequent autoimmune or idiopathic origin [11], the search for underlying causes or associations with infectious or parasitic disease, such as in CU+, can be of help in research and practice. One of the main differences with GAA, where the acute reaction points to the precise moment of the human immune system coming in contact with the nematode, is the finding of lower antibody levels in CU+ [8, 12]. 

The experience of immune features in GAA teaches us that the allergic reaction is elicited after a secondary contact with the parasite [5, 13]. The important rise in immunoglobulin levels is similar to any secondary immune reaction against invading agents. On the other hand, the exact mechanisms by which acute urticaria/angioedema or anaphylaxis is elicited in GAA, compared to other parasitic forms without cutaneous or systemic hypersensitivity reaction, are not known. Moreover, the possible pathogenesis leading to chronic urticaria in sensitized patients has not been elucidated.

Acute allergic hypersensitivity reactions have mainly been studied in the fields of food or insect allergy. Besides the necessary production of IgE induced by proteins with specific allergenic properties, several other factors are necessary to produce allergic reactions, as IgE presence is not equivalent to clinical allergy. Further measurable amounts of detectable IgE do not properly predict allergic reactions. Further relevant factors could include the production of high-affinity IgE or the ratio of other blocking immunoglobulin isotypes, such as IgG_4_ [14, 15].

Affinity and avidity studies have shown that continuous or repeated contact with antigen leads to an antibody affinity maturation [16]. This knowledge has led to studies differentiating acute from chronic infections [17–21]. 

Therefore, one approach to get more insight into the clinical relevance of specific IgE and IgG in *A. simplex*-associated allergic diseases is to assess hypothesized differences in avidity in GAA and CU+ and to associate them with the time interval elapsed since the parasitic episode and also with fish-eating habits as a possible correlation to the probability of previous subclinical parasitic episodes. 

## 2. Methods

Patients were recruited at our allergy service and included prospectively if they matched the inclusion criteria. The study protocol was approved by the University Hospital La Princesa Ethical Committee.

All patients had to display a positive SPT and detectable IgE antibodies against *A. simplex* at ≥0.7 kU/L.

GAA patients had an acute episode of acute urticaria, angioedema, or anaphylaxis of less than 48-hour duration within 24 hours after eating raw or clearly undercooked fish, and other eliciting causes have been discarded by history and allergological workup.

CU+ patients were included if they had at least twice per week recurring wheals for at least 6 weeks. Patients were not included in this study if physical stimuli were the main eliciting agents of the urticarial reaction. Other factors associated with CU were not an exclusion factor, as CU is mainly multifactorial.

Thus 16 patients with GAA and 17 patients with CU+ were included and questioned about weekly fish and monthly anchovies in vinegar sauce consumption. Further time interval was defined as the interval between serum sample and GAA episode or onset of CU symptoms.

Anti-*Anisakis *specific IgE, IgG, and IgG_4_ and total IgE were analysed by CAP-FEIA (Phadia, Uppsala, Sweden) [12]. Specific IgE and IgG against *A. simplex *whole body extract were further assessed by ELISA [22]. 

### 2.1. Avidity ELISA

Avidity ELISA was based on the dissociative method using urea as a denaturing agent [23]. Plates were coated with *A. simplex *whole body extract at 10 *μ*g/mL overnight and incubated for 30 min with 6 M urea in PBS at room temperature. Microplates were washed and blocked with BSA for 1 h. Each serum was studied in quadruplicate (2 with urea treatment and 2 without), diluted in PBS-Tween 20, BSA 0.1%, and incubated for 2 h at 37°C. After incubating and washing, two of the quadruplicate sera were treated with PBS 6 M urea for 30 min at room temperature and the remaining sera were incubated with PBS for 30 min. After washing, goat anti-human IgG and horseradish peroxidase (HRP) conjugate (BIOSOURCE, Camarillo, CA, USA) were used. For the IgE determination, a murine monoclonal antibody against an *ε*-human IgE chain (IgG1*κ*, E21A11; INGENASA, Madrid, Spain) was added and incubated, followed by a goat anti-mouse IgG (*γ*1), HRP conjugate (INVITROGEN, Eugene, OR, USA). The reactions were developed with *o*-phenylenediamine with hydrogen peroxide and read at 490 nm. The avidity index (AvIgG and AvIgE, resp.) was defined as the mean optical density (OD) of urea-treated wells/mean OD urea-untreated wells × 100.

### 2.2. Statistics

Statistical analysis was performed using SPSS ver. 15.0 for Windows. 

Median values and interquartile ranges (IQR) were calculated for total and specific IgE or IgG_4_ as well as for avidity indexes and compared by Mann-Whitney *U* test.

We performed nonparametric correlation (Spearman Rho) studies between AvIgE, AvIgG, immunoglobulins, time interval, and fish consumption.

Furthermore, a linear regression was performed separately for GAA and CU+ for IgE avidity as dependent variable. We analysed specific IgE, total IgE, IgG, IgG_4_, fish intake, and time interval as possible explaining variables with stepwise exclusion of nonsignificant variables.

## 3. Results

Mean age of the studied patients was 52.5 ± 14.3 years. 18 patients were females and 15 were males. These data did not differ in both groups.

Mean time from the acute parasitic episode to the serum sample in GAA was 5.0 (±5.2) months and mean duration of urticaria in CU+ was 27.0 (±20.8) months.

Mean number of fish meals per week was 2.7 (±1.5) in all patients without differences in both studied groups.

As expected, total IgE as well as specific IgE, IgG, and IgG_4_ was higher in GAA than in CU+ (Table 1). 

Median AvIgE in all patients was 80.3 (interquartile range 73.2–91.8), while median AvIgG was 53.9 (37.4–91.8). Table 1 shows the results when comparing both studied groups. IgG avidity was higher in GAA than in CU+ (*P* = 0.035), whereas there was a tendency to lower IgE avidity in GAA (*P* = 0.095). 

When analysing all patients, AvIgG was positively correlated with specific IgE, IgG, and IgG_4_ as well as total IgE (Rho between 0.66 and 0.71; *P* < 0.002), but AvIgE was negatively correlated with specific IgE (Rho −0.57; *P* < 0.001), specific IgG_4_ (Rho −0.38; *P* < 0.05), and total IgE (Rho 0.66; *P* < 0.001).

**Figure 1:**
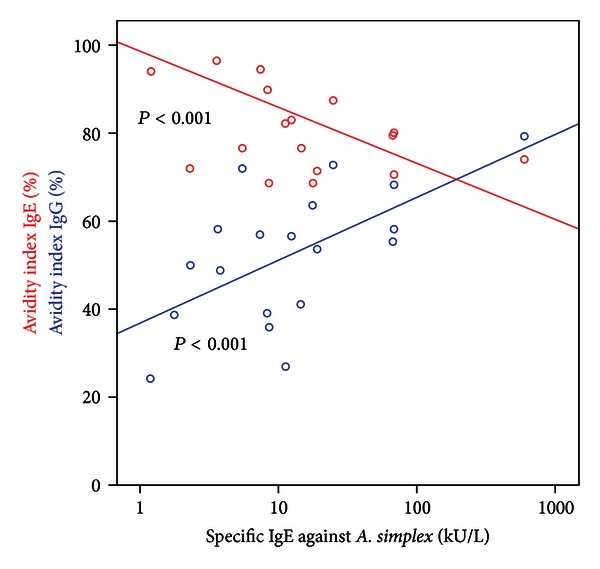
Avidity index of specific IgE and specific IgG against *Anisakis simplex* in gastroallergic anisakiasis depending on the amount of detectable specific IgE.

**Figure 2:**
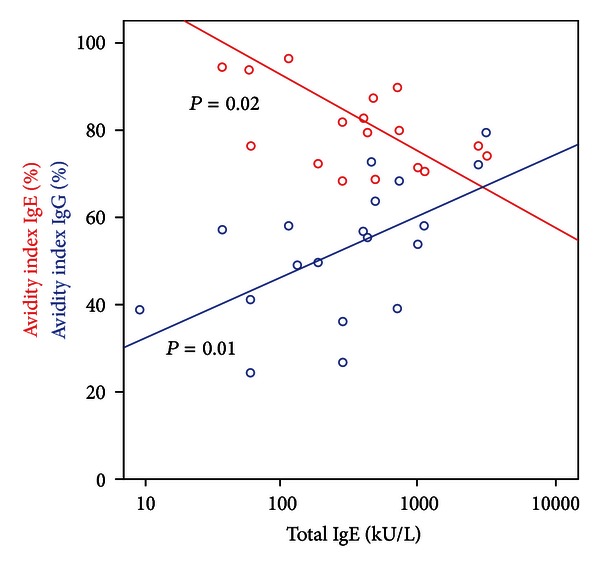
Avidity index of specific IgE and specific IgG against *Anisakis simplex* in gastroallergic anisakiasis depending on the amount of detectable total IgE.

This divergent correlation depending on AvIgE or AvIgG was mainly maintained when patients with GAA were analysed (Figures 1 and 2). Here, a further negative correlation between AvIgE and time interval could be stated (Rho −0.71; *P* = 0.02). Also, in GAA weekly fish consumption was positively associated with AvIgE (Rho 0.51; *P* = 0.05).

**Figure 3:**
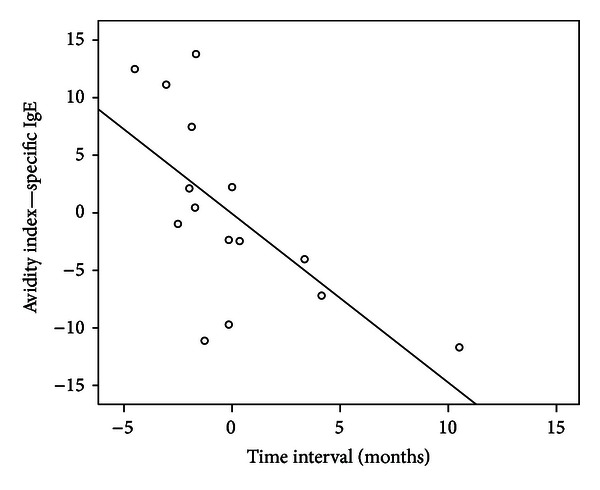
Partial regression graphic showing negative correlation of specific IgE avidity index in relationship with time interval (elapsed time from acute parasitic episode to serum sample in months).

Linear regression showed that time interval (*P* = 0.015) was the main explaining factor for AvIgE in GAA (Table 2 and Figure 3).

## 4. Discussion

 Overall, we show high avidities for specific IgE and moderate avidities for specific IgG against *A. simplex* in both studied groups. Most avidity studies have been performed in order to search for differentiating recent versus chronic infections [17–19]. When analysing our patients, we have to take into account that the positive skin prick test or the presence of specific IgE denotes a past infection by the parasite, which is always self-limiting and therefore acute. Moreover, in order to have an allergic reaction accompanying the acute gastric parasitism, as in the case of our GAA patients, they obligatorily had a previous sensitizing episode [13]. In our CU+, we do not know the time of the last parasitic episode, but lower IgE values as demonstrated in the present as well as previous studies indicate a longer time interval to the last episode [8, 24]. Thus, it is to be expected that relevant antibodies had sufficient time to affinity maturation. Whereas in chronic infection, such as by *Toxocara* or *Toxoplasma,* the continuous contact with antigens facilitates affinity maturation [18, 23], there remains however the question about the source of antigen or allergen for affinity maturation in the case of rarely occurring acute episodes of parasitism such as by *A. simplex*. 

 In a previous *A. simplex* infection model in rats, avidity tended to exceed that at primary infection for several immunoglobulin isotypes [25]. Previous studies showed that most patients with CU+ really had a past infection with *A. simplex,* which is supported also by high IgE avidities. We have no data on avidities after a primary infection with *A. simplex* in humans, as these are expected to behave subclinically or induce only gastrointestinal, but no allergic symptoms. It may be argued that in GAA at least two episodes, as proposed above, would be sufficient to induce in the second episode a higher maturation of antibodies in the context of a secondary response [13], but the otherwise longer time interval in CU+ could be responsible for the slightly higher IgE avidities. 

Whereas avidity describes the overall strength of the interaction between antibodies and their antigens, it would therefore be interesting to perform a similar study analysing affinities to already known *Anisakis* proteins or allergens. Our avidity results could be interpreted as a subset of clinically relevant IgE for the acute parasitism and a set of IgE antibodies induced by continuous fish intake (and therefore *Anisakis* contact) with possibly different avidities. In this respect, a previous study in peanut allergic subjects showed IgE : IgG avidity ratios to be higher than 1 for a total antigenic source, whereas the opposite ratio was found when analysing avidity to a major allergen Ara h2 [26]. In our present study all but one patient showed a higher AvIgE than AvIgG (AvIgE/AvIgG ratio > 1; data not shown), a fact that does not rule out opposite findings if we would have available affinity studies with known allergens.

Whereas all immunoglobulin isotypes are known to be lower in CU+ than in GAA, a fact we could also confirm for specific IgG in our study, avidities could also be interpreted when analysing the time interval between parasitism and obtaining of the serum sample. Likewise, regression analysis showed that, in GAA, time interval was the main explaining factor for IgE avidity.

Affinity is overall expected to rise in time after infection, but this probably applies to only a few relevant allergens or epitopes [26]. A possible explanation for our results could be drawn from the correlation studies depicted in Figures 2 and 3. Polyclonal stimulation after an acute parasitic episode leads to high immunoglobulin levels, and in the case of IgE, most IgE does not recognize *Anisakis* proteins [2, 13]. Even if we only focus on specific IgE production, a mixture of high-affinity and low-affinity antibodies is to be expected. As antibody levels continuously fall again after a peak at about one month, low-affinity IgE antibodies seem here to turn up over high-affinity antibodies resulting in overall lower avidity. There is still controversy about the beneficial role of low-affinity IgE antibodies. According to Xiong et al. these can compete with high-affinity IgE for binding to high-affinity IgE receptors and prevent anaphylaxis in atopic allergy [14]. Otherwise this possibility has been stated unlikely because very high levels of IgE are needed to adequately saturate IgE receptors [27].

Again, the time interval seems to be the main explaining factor. Also in this case we could speculate that, once a sensitization process has occurred, following continuous contact with *Anisakis* proteins of other specificities than those leading to the allergic episode, leads to maintaining lower affinity antibodies. This would be an explanation to the fact that patients sensitized to *Anisakis* do not react with acute type 1 hypersensitivity reactions in provocation tests with non viable *Anisakis* larvae [28, 29]. Furthermore, blocking antibodies, such as IgG_4_ included in the IgG compartment, with confirmed higher avidities in GAA could, by competing with the same epitopes recognized by IgE, prevent mast cell activation and the subsequent allergic episode, when *Anisakis* contaminated fish (nonviable or inactivated larvae) is well tolerated by patients with previous GAA. 

Interestingly, if we look at the diverging correlation of AvIgG and AvIgE with specific IgE, we can also postulate that avidity is not only the outcome of an affinity maturation process of antibodies *within* an antibody isotype, but also the outcome of a mechanism, in which probably outcompeting of an immunoglobulin isotype over another could have been resulted by high-affinity antibodies in the maturation process [30].

A possible drawback of these results is of methodological concern, as there could be some controversy about the influence of IgG antibodies on an ELISA assay in avidity studies. However, previous studies with using thiocyanate as denaturing agent showed independence of immunoglobulin levels and avidity and lack of independence between avidity indexes and immunoglobulin concentrations [26, 31]. Therefore, our finding of a negative correlation of specific IgE with avidity is not expected to be influenced by a methodological constraint. 

We did not show a direct negative correlation between AvIgE and AvIgG, but this could be due to the fact that we did not measure differential epitope affinity and the high variation of binding capacities and maturation process of antibodies between individuals [17]. In this respect it is interesting to note that differences in the generation of high-affinity IgE antibodies could be due to differences in sequential class switching versus direct class switching from IgM, although animal models and application in human allergic disease are still a matter of debate [14, 27]. The specific affinity maturation in *Anisakis*-associated allergic disease has not been studied so far, but our results could lead to a testable hypothesis, where clinically different CU+ and GAA after parasitism could be motivated by distinct class switching of specific antibodies.

## 5. Conclusions

In this first study on immunoglobulin avidities in humans affected by *A. simplex* parasitism, we could thus show a differential behaviour of immunoglobulin isotype avidities in GAA and CU+ and their dependence on fish-eating habits as well as on the time elapsed to the last parasitic episode. Further studies should address avidity studies against excretory-secretory proteins as well as affinity studies against known allergens.

## Figures and Tables

**Table 1:** Immunoglobulins and avidities in GAA (gastroallergic anisakiasis) and CU+ (*Anisakis simplex* sensitization-associated chronic urticarial).

	Total IgE (kU/L)	*Anisakis* IgE (kU/L)	*Anisakis* IgG (kU/L)	*Anisakis* IgG_4_ (kU/L)	AvIgE	AvIgG
GAA	487 (98−1142)	48.7 (9.2−326)	17.8 (12.6−42.2)	1.930 (752−15842)	78.4 (71.4−88.6)	58.2 (48.2−70.5)
CU+	288 (117−690)	5.7 (3.7−11.9)	7.8 (7.2−15.0)	0.370 (95−910)	82.4 (74.4−98.0)	49.4 (38.0−57.1)

*P* =	0.29	0.005	0.01	0.001	0.095	0.035

Values are given as median (± interquartill range) and statistical significance is given for comparison between both studied groups. AvIgE: avidity index of specific IgE. AvIgG: avidity index of specific IgG.

**Table 2:** Regression analysis for factors potentially explaining avidity index of specific IgE in gastroallergic anisakiasis.

Variable	Standardized coefficient *B *	Significance
Constant		**<0.001**
Total IgE	−0.28	*0.21 *
Time interval	−0.59	**0.02**
Fish intake	0.27	*0.23 *

Time interval explains avidity of specific IgE in gastroallergic anisakiasis. Linear regression analysis with stepwise exclusion of nonsignificant variables.

## Abbreviations

GAA: Gastroallergic anisakiasis
CU+: *Anisakis simplex* sensitization-associated chronic urticaria
AvIgE: Avidity index for specific IgE against *A. simplex *
AvIgG: Avidity index for specific IgG against *A. simplex*.
